# Editorial: Neuroepigenetics of Neuropsychiatric Disease—Hope, Success and Obstacles for Translational Findings and Applications

**DOI:** 10.3389/fnins.2022.886695

**Published:** 2022-04-01

**Authors:** Yan Jiang, Julia M. Schulze-Hentrich, Mira Jakovcevski

**Affiliations:** ^1^Institute of Brain Science, Fudan University, Shanghai, China; ^2^Centre for Rare Diseases, Institute of Medical Genetics and Applied Genomics, University Hospital and Faculty of Medicine, University of Tübingen, Tübingen, Germany; ^3^Institute of Biology II - Functional Epigenetics in the Animal Model, RWTH Aachen University, Aachen, Germany

**Keywords:** circRNA, nutrition, DNA methylation, BDNF, HDAC4, protocadherins

According to the World Health Organization, over one billion people worldwide suffer from neuropsychiatric diseases (World Health Organization, 2007). The lack of effective treatments is mainly due to our limited knowledge of the underlying pathogenesis. It has been well-accepted that besides genetic predispositions, environmental factors play essential roles in developing mental illness (Caspi and Moffitt, 2006). Indeed, clinical and animal studies have shown that many environmental factors participate in regulating mood behaviors, including chronic stress (Quello et al., 2005), major adverse life events (Corrarino, 2008), dietary factors (Owen and Corfe, 2017), drug or alcohol addiction (Lee et al., 2017; Lees et al., 2020), and endocrine disruptors (Tiffon, 2018; Rivollier et al., 2019). In addition to direct effects in adulthood, environmental insults can also influence embryonic and early brain development, thereby increasing the risk of mental disorders in adulthood (Serpeloni et al., 2019) (Figure 1).

**Figure 1:**
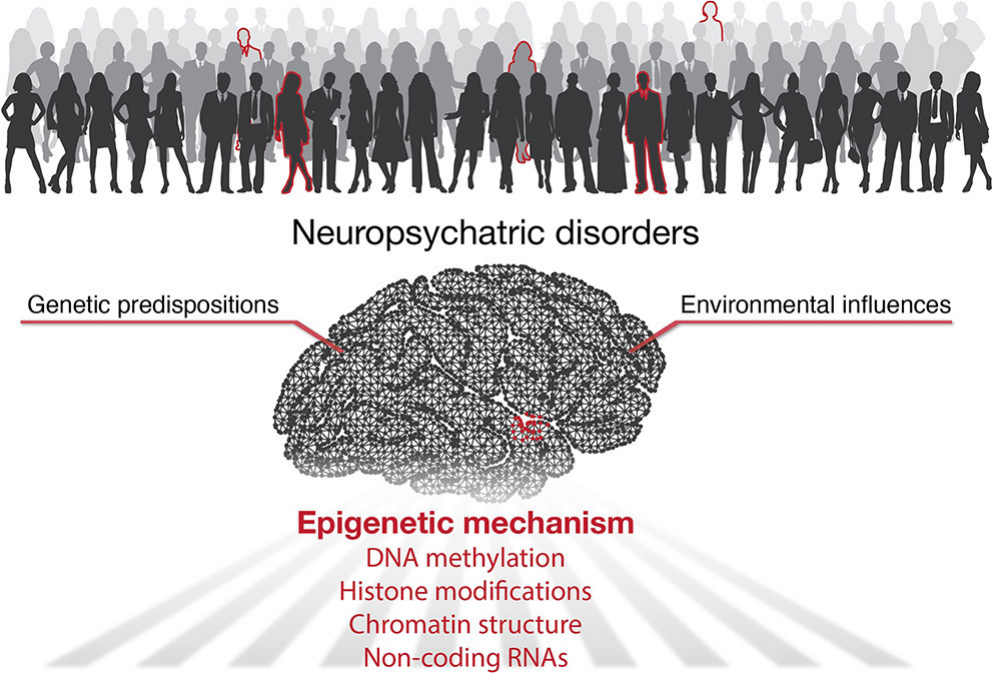
Graphical abstract shows mechanisms for development of neuropsychiatric diseases.

Accumulating studies highlighted the essential role of epigenetics for gene-environment interactions with its implications for neuropsychiatric disorders (Tsuang et al., 2004; Lin and Tsai, 2020). Epigenetics refers to heritable changes in gene expression without changing the DNA sequence of genes. Epigenetic processes including DNA methylation, histone modifications, non-coding RNAs (ncRNAs), and higher-order chromatin organization. Typically, DNA methylation and histone modifications alter chromatin accessibility or serve as docking sites to recruit other functional proteins to turn genes “on” or “off.” ncRNAs work both on the transcriptional and post-transcriptional level to affect the production and stability of mRNAs. In addition, the organization of the 3D genome, in which spatial interactions of chromatin help bring linearly distant genes and regulatory elements into proximity (such as enhancer-promoter loops), has emerged as a new epigenetic mechanism in recent years (Rajarajan et al., 2016; Sun et al., 2021). In the current Research Topic, we present seven articles exploring different aspects of epigenetics for multiple neuropsychiatric conditions (Figure 1).

DNA methylation is one of the well-studied epigenetic mechanisms and collective evidence supports its role in various aspects of brain disorders (Bakusic et al., 2017). Dysregulation of excitation and inhibition balance (E/I balance) in the brain circuits is one of the significant neuropsychological changes for many mental illnesses (Sohal and Rubenstein, 2019; Molina et al., 2020). GABAergic inhibitory interneurons are essential to keep the E/I balance in the cortex (Tremblay et al., 2016), and DNA methylation is vital for the development and function of cortical interneurons. Linde and Zimmer-Bensch discuss the role of DNA methylation and its writers, DNA methyltransferases DNMT1 and DNMT3A, in regulating essential genes for GABAergic neuronal functions, as well as genes of the endocytosis process critical for synaptic neurotransmission.

Besides perturbations in neurons, glia pathology also participates in psychiatric disorders (Cotter et al., 2002). For example, white matter lesions are observed in brains of patients with schizophrenia (SCZ) (Höistad et al., 2009). Oligodendrocytes are the myelinating cells in the central nervous system (CNS), and their disruption causes white matter (composed of myelinated nerve cells) damage. DNA methylation regulates oligodendrocyte differentiation during normal development (Moyon and Casaccia, 2017), and dysregulation of DNA methylation in myelinating glia is involved in aging and neurologic diseases (Arthur-Farraj and Moyon, 2020). Chen et al. provide new evidence for oligodendroglial DNA hypermethylation and SCZ-like behavioral deficits in adolescent mice with supply of l-methionine (met), a methyl-donor for DNA methylation. Besides oligodendrocytes, other glia cells, including astrocytes and microglia are implicated in psychiatric disorders. It will be interesting to check whether the supply of met affects other glia or maybe neurons as well, and thus contribute to the observed behavioral abnormalities.

Since DNA methylation is critically implicated in many neuropsychiatric diseases, it presents a potential target for treatment (Sales and Joca, 2016; Shirvani-Farsani et al., 2021). However, the development of specific therapeutic reagents is quite a challenge. Instead, researchers use DNA methylation to predict drug response in psychiatric disorders. Zhou et al. contribute a systematic review on published data of drug response-related DNA methylation in SCZ, bipolar disorder (BD), and major depressive disorder (MDD). Among all these studies, only the correlation between methylation at the BDNF gene locus and antidepressant effects in MDD was reproduced by multiple groups (Januar et al., 2015; Zhou et al.). Since the antidepressant effect of BDNF is well-established in animal studies (Lee and Kim, 2010), this provides evidence for using current animal models (stress-induced depressive-like behaviors in mice and rats) as valid tools for studying mental disorders. Meanwhile, cell-type specificity of epigenetic signatures may explain the limited agreement of current studies as they commonly collect complex peripheral tissue such as blood to profile DNA methylation.

Histone acetylation is another classic epigenetic mark for transcriptional activation (Hebbes et al., 1988). Histone deacetylases (HDACs) remove acetylation and thus exert transcriptionally repressive effects (Milazzo et al., 2020). Previous work from Cui et al. (2013) identified a missense mutation in *HDAC*^A786T^ that increases the risk for eating disorders (EDs). In the current topic, Davis et al. generated a transgenic mouse model carrying this mutation and revealed gender- and circadian-related behavioral deficits associated with EDs. This work provides evidence for *Hdac4* in EDs and generates a valuable model for future studies on the neuropsychiatric basis of EDs.

In addition to changes at the level of genomic DNA and histone modifications, long non-coding RNAs (ncRNAs) serve as another layer of epigenetic regulatory mechanism (Cao, 2014). Circular RNAs (circRNAs) are a novel set of ncRNAs and highly expressed in the CNS, particularly important for regulating synaptic functions (Kocerha et al., 2015) and involved in psychiatric disorders (Yoshino and Dwivedi, 2020). Paudel et al. reported gender-specific changes of circRNAs after prenatal alcohol exposure (PAE) in the embryonic brain, and the expression of some circRNAs was correlated with neuronal and glial gene expression.

MDD is becoming one of the most severe health problems globally (Otte et al., 2016). Besides chronic stress that is well-accepted as top one risk factor for MDD (Breslau and Davis, 1986), other environmental factors, including dietary influences, contribute to the neuropathogenesis of depression (Firth et al., 2020). In this topic, Aly and Engmann reviewed current knowledge on some of these dietary factors, such as vitamins, fatty acids, and minerals, associated with MDD and may serve as potential antidepressant targets. Many nutritional factors are also reported to affect epigenetic events, especially for enzymes involved in DNA methylation and histone modifications (Maugeri and Barchitta, 2020). For example, vitamin B3 is one of the precursors for nicotinamide adenine dinucleotide (NAD), the essential cofactor for type III histone deacetylases (HDACs). In addition, S-Adenosyl Methionine (SAM), a methyl donor for DNA methylation and histone methylation, can be found in most dietary proteins. Vitamin B12 serves as a cofactor of methionine synthase, and vitamin B9 (also known as folic acid) participates in the vitamin B12-mediated SAM metabolic pathway once biologically activated *in vivo* (Crider et al., 2012). It would be interesting to provide direct evidence for connections between these essential nutritional components, epigenetic events, and depressive behaviors.

In recent years, the 3D configuration of chromatin has been recognized as one of the important strategies of epigenetic regulation. The linear genome is highly compacted and well-organized *via* chromatin loop interactions inside the nucleus (Zhao et al., 2019). Disturbance of the 3D genome is now considered an intriguing mechanism for psychiatric disorders (Rajarajan et al., 2018). Clustered protocadherin genes (*cPcdh*) encode a subfamily of cell adhesion molecules, predominantly expressed in the brain. The combination of different *cPcdh* expressions serves as an identity code for individual neurons (Wu and Maniatis, 1999). The *cPcdh* locus is one of the best-studied examples for regulating stochastic and combinatorial expression patterns *via* 3D chromatin organization (Wu and Jia, 2021). Previous seminal studies of Dr. Qiang Wu's group reported complex regulatory mechanisms for *cPcdhs*. More importantly, it described the role of chromatin organizer CTCF for the 3D chromatin organization at this locus (Wu et al., 2020). In the current topic, Jia and Wu provided a detailed review summarizing the biological function and regulatory mechanism for the *cPcdh* locus and its implications for various neuropsychological disorders.

The work presented in this Research Topic spans a variety of neuropsychiatric conditions, covering most of the important epigenetic mechanisms, including DNA methylation, histone modification, circRNAs, and higher-order chromatin organizations. Together, they would advance our understanding of neuroepigenetics in mental disorders and inspire the hope and efforts for successful translation to clinical care in the future.

## Author Contributions

All authors listed have made a substantial, direct, and intellectual contribution to the work and approved it for publication.

## Funding

This work was funded by grants of National Natural Science Foundation of China (No. 81971272) and Science and Technology Commission of Shanghai Municipality (No. 19ZR1405400 to YJ).

## Conflict of Interest

The authors declare that the research was conducted in the absence of any commercial or financial relationships that could be construed as a potential conflict of interest.

## Publisher's Note

All claims expressed in this article are solely those of the authors and do not necessarily represent those of their affiliated organizations, or those of the publisher, the editors and the reviewers. Any product that may be evaluated in this article, or claim that may be made by its manufacturer, is not guaranteed or endorsed by the publisher.
